# Influence of Surface Treatment and Protracted Ageing on the Shear Bond Strength of Orthodontic Brackets to Two Digitally Fabricated (Milled and 3D-Printed) Polymethacrylate-Based Provisional Crowns

**DOI:** 10.3390/polym17050699

**Published:** 2025-03-06

**Authors:** Nisreen Nabiel Hassan, Khurshid Mattoo, Atheer Khawaji, Hanan Najmi, Almaha Sadeli, Ahid Amer Alshahrani, Abeer Ali Qahtani, Abdullah Hasan Alshehri, Mai Almarzouki, Mohammed E. Sayed

**Affiliations:** 1 Department of Restorative Dental Sciences, College of Dentistry, Taibah University, Madinah 41477, Saudi Arabia; nnhassan@taibahu.edu.sa; 2 Department of Prosthetic Dental Sciences, College of Dentistry, Jazan University, Jazan 45142, Saudi Arabia; kmattoo@jazanu.edu.sa; 3 Intern Clinic, College of Dentistry, Jazan University, Jazan 45142, Saudi Arabia; atheer.khawajix2@gmail.com (A.K.); hanan.najmi111@gmail.com (H.N.); sadeli.almaha@gmail.com (A.S.); 4 Department of Dental Technology, College of Applied Medical Sciences, King Khalid University, Abha 62529, Saudi Arabia; aalshahrani1@kku.edu.sa; 5 Department of Restorative Dental Science, Taif University, Taif 26571, Saudi Arabia; abeer.a.qahtani1@gmail.com; 6 Department of Prosthodontics, College of Dentistry, King Khalid University, Abha 62529, Saudi Arabia; abhalshehri@kku.edu.sa; 7 Department of Restorative Dentistry, Faculty of Dentistry, King Abdulaziz University, Jeddah 21589, Saudi Arabia; mzalmarzouki@kau.edu.sa

**Keywords:** three-dimensional printing, computer-aided design, computer-aided manufacturing, orthodontic bracket, temporary dental restoration, dental crowns, tetrahydrofurfuryl methacrylate

## Abstract

This study determined the influence of surface treatment and protracted ageing on the shear bond strength (SBS) of orthodontic brackets bonded to CADCAM (milled) and 3D-printed polymethylmethacrylate (PMMA) provisional crowns (PCs). Eighty disc-shaped specimens [forty milled (CopraTemp WhitePeaks) [group (Gp) M] and forty printed (Asiga DentaTooth) (Gp P)] were divided into eight subgroups (Gp) based on surface treatment [no treatment (control) (Gp MC and Gp PC), coarse diamond (Gp MCD and Gp PCD), fine diamond (Gp MFD, and GP PFD) and sandblast (Gp MSB and Gp PSB)]. Orthodontic brackets were bonded (Assure Plus, Transbond XT), thermocycled (2200 cycles), and tested for SBS and failure (Adhesive Remnant Index) (ARI). Statistical tests included analysis of variance (ANOVA); Kruskal–Wallis (ARI ranks); and post hoc (Tukey, Dunn, and Bonferroni) for determining group differences at predetermined probability *p*-values less than 0.05. SBS was significantly increased in Gp MSB (15.51 Mpa) and Gp PSB (14.11 Mpa), while the coarse diamond subgroups yielded the lowest mean SBS values [Gp MCD (11.28 Mpa) and Gp PCD (11.62 Mpa)]. The SBS of subgroups MFD, MSB, PCD, and PSB showed significant differences from those of their respective controls (Gp MC and Gp PC). Low ARI scores were observed in Gp MC (0.40) and Gp MSB (0.80), while higher scores were observed in Gp PCD (2.10). Both milled and printed PCs fulfil the clinical criteria of the minimum SBS for orthodontic brackets for long-term use. However, milled PC has better SBS and low ARI scores, which make it more clinically feasible for orthodontic treatments.

## 1. Introduction

A provisional crown (PC) may be encountered or requested to facilitate an orthodontic treatment. Interdisciplinary pre-restorative mouth preparations, orthodontic root extrusions, complete occlusal rehabilitations [1,2], intradisciplinary preventive orthodontics in deciduous or mixed dentitions, and bonding brackets in conditions like amelogenesis imperfecta, creating spaces for implant placements [3,4], treatments require a PC placement to initiate respective treatment goals [5]. The purpose of PC during orthodontic treatment is to provide the necessary functions (protection, stabilisation, mastication, and aesthetics) of a lost/missing tooth, besides aiding the orthodontist in determining the orthodontic-related aesthetic outcome of aligning teeth properly [6]. During ongoing orthodontic treatment, the PCs plays a major role in the application of forces, thereby contributing to the orthodontic treatment outcome [7]. Orthodontic treatment durations are generally long, 6 months to 3 years; therefore, the PC used must serve for long time periods. [4] Clinically, shear forces are the most destructive forces that affect an orthodontic bracket, with tensile and torsional forces having little impact on debonding [4,8]. For orthodontic tooth movement, different clinical forces are required to be applied, which range from 35 to 60 g for extrusion and 70 to 120 g for tooth translation [9]. To affect such an application, the bond strength between the orthodontic bracket and natural tooth structure has been recommended to be at least in the range of 6 to 8 MPa [9]. Critics, however, have questioned the threshold values for clinical application because they were determined through in vitro tests [10] and have cautioned against inferring such low values to levels that are “clinically admissible or adequate” [11]. Because this “threshold bracket bond strength” ignores therapeutically relevant variables (such as pH, temperature, humidity changes, fatigue-related adhesive, and microbiological degradation), critics are able to support their assertions [1,2,11]. This is authenticated by reports that found in vivo-aged bond strength is significantly lower than in vitro-aged bond strength [8,12]. Besides the differences between in vivo and in vitro environments, there are other factors that influence this bond strength, namely provisional material type [6], adhesive used [13], storage time [14], and ageing [12]. Ideally, the most appropriate bond strength is the one that maintains bracket positions during entire orthodontic treatment; withstands all biomechanical forces from teeth and surrounding musculature; does not fail due to a changed oral environment pH and bacteria; and, more importantly, allows debonding (removal) without causing any type of damage to the bonded surface (tooth, PC, or permanent crown) [15].

As per the FDA regulations, there are four key dental materials that serve safely for temporary restorations: polymethyl methacrylate (PMMA), polyethylmethacrylate (PEMA), bisacryl composite, and light-cured composite resin [16]. Bisacryl composites have been mostly recommended [low exothermic reaction, high strength, less shrinkage, enhanced marginal adaptation, and colour stability] during the last two decades [1,5,6,17], while PMMA allows superior mechanical and optical properties at lower cost besides multiple arrays of fabrication techniques [direct, indirect, indirect direct, milling, and printing] [6,16]. Its main drawbacks [shrinkage, exothermic polymerization, and degrading matrix] have been overcome by application and the introduction of computer-aided design and computer-aided machining [CADCAM], which uses either milling or printing to fabricate PC from either a pre-polymerized puck or resin ink [18]. The long-term PCs have traditionally remained the PMMA resin, preferably the one that has been cured by heat, to decrease the residual monomer content that undergoes degradation [19]. Depending upon the material type, various surface treatments (mechanical or chemical) have been experimented with to improve the shear bond strength (SBS) of temporary or provisional materials (dichloromethane solvent [20], acid etching [21], and sandblasting [22]), as well as permanent crown materials (surface grinding [23] and laser [24]). Drawbacks associated with traditional materials and techniques of fabrication from acrylic and composite resins have been largely overcome with digital oral impressions and their respective applications in CADCAM milling and three-dimensional (3D) printing. Both milling and 3D printing have found applications in most of the restorative materials, such as resin composites; polymer resins (PMMA, PEMA, and PEEK); dental ceramics (zirconia, lithium disilicate, and leucite); and metals (titanium, stainless steel, and chromium cobalt alloys) [25]. Both technologies have paved the way for providing temporary and permanent restorations, models and casts, implant templates, and maxillofacial prostheses [26]. Milled resin has been reported to possess higher strength and durability than conventional self-cured PMMA when used for PCs after ageing [27,28]. Besides their aesthetic (optical) and mechanical properties, they have also been superior to conventional handmade resin restorations [29]. The chief drawback of this technology is its high cost, compounded by increased waste of raw materials and microscopic wear and tear of both milling tools and material, besides producing noise and heat during production [30]. On the other hand, 3D-printed interim restorations have gained popularity. Presently, 3D printing techniques have seven general industrial applications, out of which, four have been successfully used in dentistry [material jetting, stereolithography, digital light processing, and material extrusion] [31]. Differences in technologies are either due to the material used or layer-building methods of creating a three-dimensional object. The chief advantages of 3D printing are its minimal material use, which reflects directly on its low cost, fabrication of numerous items, ease of operation, and higher resolution to produce intricate details (occlusal anatomy) [32]. Its disadvantages, like polymerization shrinkage, resin ink and machine cost, and surface roughness, have been overcome by the calibration of printers, smaller portable machine availability, and affordability [16,25]. Lee et al. found that their 3D-printed PCs had a better internal crown fit than milled PCs [33]. Peng et al. observed that 3D-printed PCs had a better marginal fit with less internal discrepancy than manually prepared PCs [34]. Park et al. reported 3D-printed resin material to have wear resistance that equalled most other self-curing temporary resin materials [35], thus making them a good alternative to long-term PCs. Tahayeri A et al. improved 3D printing by studying printer optimisation, which included printer orientation and print thickness in PCs. He observed that the final properties of the PCs are significantly influenced upon the pre-optimization of printers for each material used (ink) [36]. Al Dwairi et al., while comparing three different brands of printed resin against PMMA resin (heat cure), found one of the printed resins (Asiga Dentamodel) to have a significantly low surface roughness (0.19 ± 0.03 μm), which improved its long-term durability in terms of optical and mechanical properties [37]. Di Fiora et al. reported 3D-printed PMMA to have lower bacterial adhesion than heat-polymerized PMMA but more than milled PMMA [38]. Shin JW et al., while evaluating a 3D-printed PC material (NextDent), reported higher staining only with different food colourants [30].

Few studies have investigated the shear bond strength between orthodontic brackets and milled provisional crowns [39,40]. Haber D et al. searched for the best mechanical, chemical, and/or combination surface treatments for CAD/CAM PCs. He concluded that orthophosphoric acid etching that is performed on natural crowns should not be done on milled PCs, besides concluding that mechanical surface treatments (diamond bur and sandblasting), along with chemical treatment (plastic conditioner), produced SBS equivalent to those with natural teeth [39]. Goracci C. et al., in their study on the effects of various surface treatments (medium and fine grit diamond bur and universal adhesive) on SBS between metal brackets and PMMA CADCAM PCs, found medium grit and universal adhesive to significantly increase SBS [40]. Sayed ME et al. reported that 3D-printed PCs were more colour stable than milled PCs when cemented with clear cement, thus making 3D-printed PCs a good aesthetic choice for long-term provisionals [16]. Any type of mechanical or aesthetic failure of PCs while undergoing orthodontic treatment has not only serious implications for the therapeutic effectiveness of the ongoing orthodontic treatment but has also been reported to have financial implications and replacement concerns [41]. Therefore, in the quest to search for an ideal long-term PC, this study was aimed at investigating the comparative differences between milled and 3D-printed PC materials after undergoing similar mechanical and chemical surface treatments and recommended ageing that qualifies for long-term PCs. The objective of the study was to identify the most appropriate provisional long-term restoration that should be chosen for restoring teeth while undergoing orthodontic treatment. The study hypothesises that there will be significant differences in the SBS between the two materials and that surface treatment and ageing will decrease the SBS. Alternately, the null hypothesis would state that there will be no differences between the materials and their respective surface treatments or ageing processes.

## 2. Materials and Methods

Materials: A brief list of the main materials and instruments used in the study are listed in Table 1.

**Figure 1:**
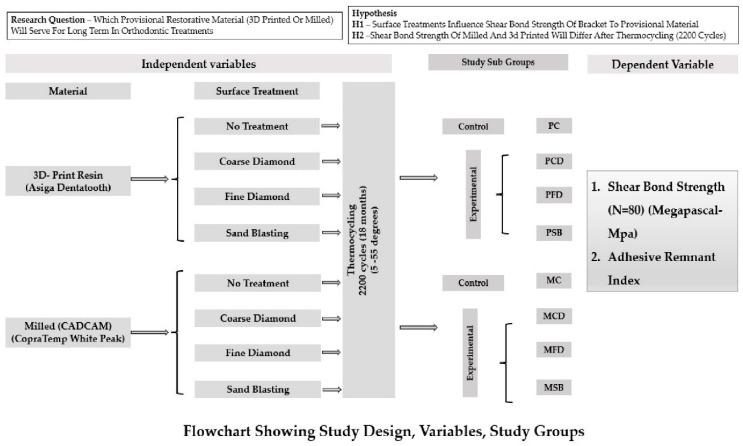
Flowchart showing the overall study design, the independent and dependent variables, and the study groups.

Study Design: This in vitro study followed a comparative (control/test) approach to experimentation that was conducted in the first two quarters of the year 2024. The study was conducted in two distinct phases, which included the preliminary phase of preparing samples and exposing them to intervention, while the final phase involved testing and data collection. The study flow chart showing the independent and dependent variables with their respective experimental interventions is presented in Figure 1.

Operational Definitions: In Ref. [42], the term ‘adhesive failure’ was defined as the bond failure at the interface between two different materials because of forces (tensile or shear). The term ‘cohesive failure’ was defined as the failure of a bond within a dental material as a result of shear or tensile force. ‘SBS’ implicates the maximum force that an adhesive joint could tolerate before failure or fracture. ‘Shear stress’ is the internal-induced force that opposes the sliding of one plane on an adjacent plane or the force that resists a twisting action [42].

Sample Preparation, Selection, and Grouping:

Sample Size: The study design included two main groups with four subgroups in each group, making a total of eight subgroups (two controls and six experimental). Based on this study design, an effect size (variable relationship) of D2 = 0.28, a power assumption (probability value) of 80%, and a type 1 error (incorrect hypothesis rejection) rate of 0.05, the total samples required for the total study was calculated using the formula (N = 2 σ^2^ × (Z α + Z β) 2/2) using Nquery software (v7.0; Informer Technologies, CA, Los Angeles, USA) [43], which came out to be 80 specimens, with each group having 10 specimens. An additional 2 specimens per group were kept as replacements for defective or damaged ones.

Specimen preparation (Table 1): Two different digitally fabricated provisional material types were investigated as independent variables in this study: 3D print resin (Asiga DentaTooth, Alexandria, Australia) and milled (CopraTemp, White Peaks, Wesel, Germany). The common and financially viable materials, instruments, and machines with their respective features and specifications used in this study are presented in Table 1. A disc with specific dimensions (10 mm diameter/15 mm height) was designed by scanning [desktop scanner (3Shape A/S Holmens Kanal 7 1060 Copenhagen/Denmark)] a previously fabricated stainless steel metal die with similar dimensions. The scanned data of the specimen design was then transferred by respective software for producing milled (EXOCAD, GmbH, Darmstadt, Hessen, Germany) and 3D-printed (Asiga Composer software, version 2.0 specimens. CADCAM (milled) specimens manufactured from PMMA resin discs (CopraTemp WhitePeaks Dental Solutions GmbH) (Shade A3) (standard size 98/14) on a 5-axis dry milling machine (DWX-52D Series, Roland DGA, Irvine, CA, USA) using an operating speed of 6 to 1800 mm/min and a spindle speed of 6000 to 30,000 rpm [44]. High-speed cutting tools (stainless steel, 1 mm/2 mm wide, 5° edge rake angle, 2° clearance angle) milled the PMMA resin discs. Forty discs were fabricated for this study group that represented milled (code M) specimens. Specimens with a crack or surface defect were isolated, discarded, and replaced with another one. The milled specimens were then finished using the manufacturer-recommended kit (K0330 PMMA, KP, Brasseler, Savannah, GA, USA) and then polished with a polishing slurry (Pumice Fine; Benco Dental) for 90 s [45]. For specimens in the 3D print (Code P) group, the similar specified disc-shaped specimens were printed using the manufacturer-recommended digital light processing 3D printer (Asiga Pty Ltd., Alexandria, Australia) and resin ink (Asiga DentaTooth, Alexandria, Australia) (Shade A3). Before printing could be achieved, the mandatory optimisation of the printer for various parameters was conducted. These parameters included accuracy and precision, surface finish, resolution (62 μm pixel size), and build volume (X, Y, Z = 119 × 67 × 76 mm) (4.68 × 2.63 × 3 inches) [16,36]. The printing accuracy was determined by measuring the dimensions of the printed sample against the CADCAM-designed specimen dimensions using a calibrated calliper (digital, ±0.1 mm) (Mitutoyo Corporation, Tokyo, Japan). After printing at a layer thickness of 100 μm, each disc was washed with 98% pure fresh isopropyl alcohol (IPA) in a well-ventilated area. A pre- and post-wash bath in an ultrasonic cleaner was performed for 2 min. Each specimen, after washing, was left over for 30 min, ensuring the specimen became alcohol-free. The specimens were then cured with light (wavelength 385 nm), the intensity of which was first determined with a power meter (Molectron Coherent, Santa Clara, CA, USA), placed inside the chamber (NK Optik Otoflash G171) (2000 flash). After curing, the cooling of the specimens was followed by a further exposure to 2000 flashes (totalling 4000 flashes, 2 times 2000 flashes on each side). Once cured, the specimens were soaked in fresh water at room temperature for 60 min. The finishing and polishing of 3D-printed resin discs was accomplished using routine rotary finishing and polishing procedures for PMMA resin, which were similar to those used for milled specimens. Before proceeding to the next step, the final thickness of each specimen was confirmed with a digital calliper (Mitutoyo Corporation, Tokyo, Japan), followed by ultrasonic cleaning (Eltrosonic Ultra Cleaner 07-08; Eltrosonic GmbH, Brasseler, Savannah, GA, USA) for 10 min to simulate the clinically used protocol for PC restorations. Forty disc-shaped specimens were thus assigned to the 3D print group (GP P), with further subdivision based on surface treatment. Each specimen was encased in a plastic ring that held the self-curing resin that attached each specimen firmly to the plastic ring to ease the application of forces during shear testing.

Experimental Intervention

**Figure 2:**
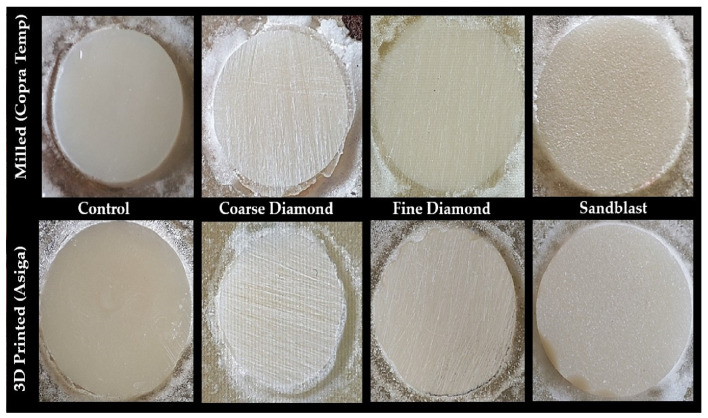
Figure showing the disc-shaped specimens of two main groups: milled and printed and their respective subgroups based on mechanical surface treatment. (from **left** to **right**) Control—no treatment, coarse diamond—180 grit sandpaper, fine diamond—320 grit sandpaper, and sandblast (50 μm Al_2_O_3_).

Surface Treatment (Mechanical) (Figure 2): The two main groups, Gp M and Gp P, were further subdivided into four subgroups, depending upon mechanical surface treatment control (no treatment), CD (coarse diamond), FD (fine diamond), and SB (sandblast), thus yielding eight subgroups, with four subgroups in the milled group [milled control (Gp MC), milled coarse diamond (Gp MCD), milled fine diamond (Gp MFD), and milled sandblast (Gp MSB)] and four in the printed group [(Gp PC), printed coarse diamond (PCD), printed fine diamond (PFD), and printed sandblast (PSB)] (Figure 2). The selection of surface treatment was based on the commonly clinically available amenities to do so. A 180-grit sandpaper simulated a coarse (medium-grit) diamond bur, while a 320-grit sandpaper simulated the surface treatment of a fine-grit diamond bur. Both sandpapers were used in wet conditions at 1.3 Newtons for 10 s [46]. Sandblasting of specimens in both groups was performed by an intraoral sandblaster (Microetcher IIA, Danville Materials, CA, Los Angeles, USA), which was filled with 50 μm of alumina oxide (Al_2_O_3_) and operated from a distance of 10 mm perpendicular to the surface with 3 bar pressure for 15 s.

Surface Treatment (chemical): Eighty stainless steel prefabricated orthodontic brackets for maxillary central incisors (Damon2; ORMCO, Glendora, CA, USA) were divided into 8 subgroups (2 control and 6 experimental). The average surface area of the bracket base, as reported by the manufacturer, was verified through a digital calliper (Mitutoyo Corporation, Tokyo, Japan). A random set of 20 brackets was measured, which provided a mean-value of the surface area of the brackets (11.01 mm^2^). To ensure standardisation of bonding, all specimens from both groups were bonded by an all-surface bonding resin (Assure Plus, Itasca, IL, USA), which is a hydrophilic, light cure bonding agent. One coat of bonding resin was applied to each specimen using a brush, which was thinned and dried out by a stream of dry air. As per the manufacturer’s recommendations, the light curing of the adhesive is recommended only if the resin that attaches the bracket is a dual cure, and in the case of the bracket resin being a light cure, then both can be light-cured together. At the same time, a small amount of Transbond XT resin paste was placed on the SS bracket base and then seated lightly against the predetermined specimen centre. A transparent thermoplastic mould that was customised to the specimen outer ring allowed standardised placement of all orthodontic brackets in the same position. Once the bracket was adjusted to its final position, it was firmly seated using a scaler tip (KaVo Perio Tip number 8, length: 38 mm, weight: 1.2 g). The same instrument tip was also used to remove the excess from the periphery of the bracket base with gentle pressure. The two were then light cured with an LED curing light (Ortholux Luminous; 3M Unitek; output: 1600 mW/cm^2^). For all specimens, the light cure tip was placed in a standard position (10 mm away) and at a standard time (24 s–12 s on two predetermined sides). A single operator performed the bonding procedure for all specimens.

Ageing (thermocycling): Artificial ageing was carried out in the laboratory using a thermocycling machine (SD Mechatronik, Bayern, Germany) that was programmed to expose the specimens to 2200 cycles (equivalent to 20 months of clinical orthodontic treatment or in vivo exposure) [39,47]. The main features of the cycle were immersion for 30 s in baths of cold and hot water, which temperatures were 5 and 55 degrees, respectively, with an interval of 10 s in open air after each relative exposure.

Measures, Data Collection, and Data Analysis:

All specimens were tested for bond strength by fastening them to the clamp (lower jaw) of a universal testing machine (Instron 5965, Instron Corporation, Norwood, MA, USA). After fastening the specimens, the debonding steel rod that has a flat end was adjusted to point at the junction between the bracket and the specimen (specimen base parallel to the force direction), with the rod moving at a crosshead speed of 1 millimetre per minute.

Shear Bond Strength: The load that was required to debond each specimen was noted in newtons, and the bond strength was later expressed in megapascals (dividing the load by the mean surface area of the brackets).

Adhesive Remnant Index (ARI): All debonded specimen surfaces were examined under an optical microscope (optical, magnification 20×) (Amscope, Savannah, GA, USA). The failure of the bracket on the specimen was then observed and analysed using the adhesive remnant index (ARI) [48]. The index provides the failure in the form of scores (0: no leftover adhesive, 1: less than 50% of leftover adhesive, 2: more than 50% of leftover adhesive, and 3: all adhesive leftover with distinct bracket mesh marks). This was achieved by dividing the area into four equal sections, each representing one-fourth or 25 percent of a total of one. Three failure categories were recognised under the microscope: adhesive failure, which was debonding at the adhesive/substrate interface; cohesive failure, when failure occurred within the adhesive; and mixed failure, which was a combination of these two or a partial adhesive present with either the substrate or the bracket. Clinical feasibility was assessed by the amount of adhesive left over the surface, indicating additional clinical time for its removal.

Statistical Analysis: Raw data were entered into a Microsoft Excel sheet, where it was first visualised, refined, corrected, and then coded. For data analysis, the coded data were loaded onto the statistical package for social sciences software SPSS (version 24, IBM, Armonk, NY, USA), while the application was run on a laptop computer [Lenovo CT45BG7, Windows 10 Pro].

Shear bond strength: The bond strength was expressed by the machine in newtons (N) which was converted to megapascals (Mpa), by dividing the load in newtons by surface area of the orthodontic bracket. The data of each subgroup was first run for a normality test (Shapiro–Wilk) to analyse the data distribution, followed by testing for homogeneity of group variances (Levene test). A one-way analysis of variance test determined the independent relation between variables of different subgroups, while a two-way analysis of variance test (ANOVA) confirmed the interaction between the studied subgroups. Tukey’s HSD (Honestly Significant Difference) post hoc pairwise comparison test was used to analyse the differences in the sample SBS means between various subgroups of the milled and printed PC groups. For statistical analysis, the probability ‘*p*’-value was considered to be significant if the difference was below or equal to 0.05 (*p* ≤ 0.05).

ARI Scores: Overall bond failure within a particular group was expressed in frequency percent to delineate the major type of failure for that group. The scores of the adhesive remnant index were assessed for statistical significance (between group differences) in relation to the amount of adhesive left over the specimen surface. The Kruskal–Wallis ANOVA (non-parametric) rank test (determine the significance of differences between the ranks of two or more groups) was applied to the obtained median ARI scores. This was followed by the post hoc Dunn’s multiple range test after Bonferroni correction. For statistical analysis, the probability ‘*p*’-value for the Kruskal–Wallis test was *p* ≤ 0.05, while the corrected alpha (α) using Bonferroni correction in the post hoc test was calculated to be *p* ≤ 0.001786 [Corrected α = α/m = 0.05/28 = 0.001786].

## 3. Results

Shear bond strength: Table 2 presents the influences of various surface treatments and the comparative differences in means of SBS obtained between SS orthodontic brackets and two different types of PC materials. The results obtained are the SBS of specimens that were bonded using different surface treatments and then aged, while the bond strength tests were performed after the completion of ageing. In the absence of surface treatment, a higher SBS strength was observed in milled PCs (m = 13.24 ± 0.94) as compared to 3D-printed PCs (m = 11.50 ± 1.8), indicating that the protracted ageing affected milled less than printed PCs. Among different types of mechanical surface treatments, sandblasting produced higher SBS in both milled (m = 15.51 ± 0.89) and 3D-printed (m = 14.11 ± 0.87), with both subgroups having the highest SBS in their respective groups. Mechanical surface treatment using a fine diamond bur produced significantly higher SBS in both milled and 3D-printed groups as compared to the coarse diamond [milled (13.76 ± 1.26) and printed (12.76 ± 1.22). Both milled (11.28 ± 0.53) and 3D-printed (11.62 ± 1.25) PCs produced the lowest SBS when they were mechanically prepared with a coarse diamond bur. The surface treatments with coarse diamonds reduced the SBS from the baseline (control) values for a milled group [control 13.24 ± 0.94 to 11.28 ± 0.53]. Both independent (one-way ANOVA) and interactive (two-way ANOVA) tests showed that significant differences existed between various subgroups of milled and 3D-printed groups (Table 2). The two-way ANOVA represents the significance of interactions between various subgroups when two or more variables are statistically analysed. Post hoc (Tukey’s HSD) pairwise comparison test results are shown in Table 3, which reflects the pair of subgroups that differed from other subgroups. Within the milled group, the mean values of two subgroups (MSB and MCD) differed significantly from the control group. The differences between the milled subgroups were significant between Gp MCD and Gp MFD and Gp MCD and Gp MSB, thus indicating that the bond strength using coarse diamond bur was inferior compared to fine diamond and sandblasting surface treatments. There was also a significant difference between the mean values of Gp MFD and Gp MSB, indicating that sandblasting produces changes in the surface that significantly increase the SBS as compared to all other mechanical surface treatments. Within the 3D-printed group, the subgroup that showed differences from the control group was only Gp PSB, while, between subgroups, Gp PCD differed significantly from Gp PSB, while no differences existed between Gp PCD and Gp PFD or Gp PFD and Gp PSB, thus indicating that, for printed PCs, the sandblast surface treatment was the only treatment that was more effective after protracted ageing.

**Figure 3:**
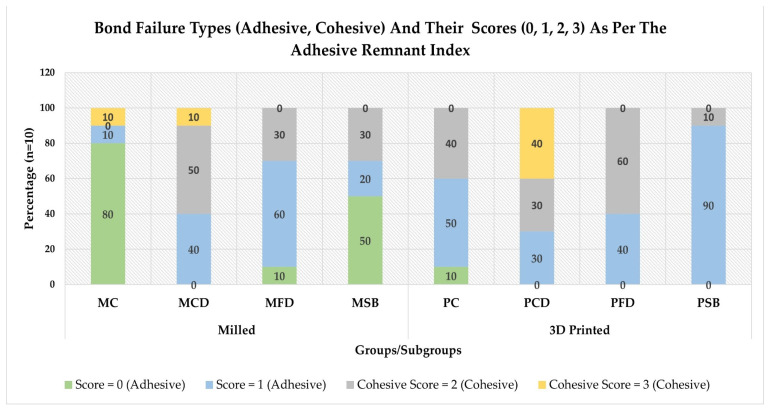
Comparative frequency distribution (in percent) of various bond failure types (adhesive and cohesive) and their scores (0 = no adhesive, 1 = ≤50%, 2 = ≥50%, and 3 = all adhesives) as per the adhesive remnant index, in specimens of various subgroups of milled And 3D-printed provisional crowns.

Adhesive Remnant Index Scores: The type of adhesive failure was assessed by the scores obtained from the ARI, with each specimen being scored from 0 to 3, depending upon the amount of adhesive left. The frequency distribution of different scores obtained in various subgroups and their respective obtained scores are presented in Figure 3. According to the index, scores 0 and 3 show purely adhesive and cohesive failures, respectively, while scores 1 and 2 show mixed failures, showing either less than or more than 50 percent of the leftover adhesive. The amount of adhesive left over the specimen surface indicates the efficiency of the surface treatment. Most adhesive failures were observed in Gp MC (80%), while most cohesive failures were in Gp PSB (90%) after thermocycling. The frequency distribution graph shows that sandblasting showed a higher number of samples with scores 0 and 1 (no or less than 50% adhesive left) over the surface for both milled (70%, n = 10) and 3D-printed (90%, n = 10). Higher cohesive failures occurred when coarse diamond bur was used to create surface irregularities for the printed group (40%, n = 10). The Kruskal–Wallis (one-way non-parametric) rank test results are presented in Table 4, showing statistically significant differences in the ARI mean rank scores among the subgroups of milled and 3D-printed PCs (*p* ≤ 0.05). The lowest mean rank score for the ARI was observed in Gp MC (17.4), while the highest mean rank score was in Gp PCD (59.35). The post hoc Dunn’s multiple range test after Bonferroni correction (*p* ≤ 0.0017) in the respective groups shows that, in the milled group, only Gp MCD’s rank differed significantly from its control group, while, in the printed group. there were no differences between any of the subgroups with its control (*p* ≤ 0.0017) (Table 5). Between the two main groups, milled and printed, Gp PCD and Gp MFD were the only two subgroups that differed from the mean of Gp MC (*p* ≤ 0.0017).

## 4. Discussion

This study investigated the influence of various surface treatments on the SBS of a stainless steel orthodontic bracket made of two digitally milled and printed PC restorative materials after undergoing a long-term ageing that clinically represents a period of 20 months, which is equal to one and a half years. The results from the study showed that both sandblasting and fine diamond surface treatment on either milled or 3D-printed crowns can be used as long-term provisional crowns during orthodontic treatment without the risk of debonding failure, since both materials fulfil the threshold of bond strength, as determined during in vitro conditions [6 to 8 MPa]. Coarse diamond surface treatment may not suffice for increasing the bond strength, since it decreases the bond strength in milled PCs while increasing minimally in printed PCs. Significant differences in SBS were observed between the two PC materials (milled PMMA and 3D-printed methacrylate-based light-cured resin) with no treatment (control), with PMMA blanks having higher SBS (m = 13.24) than 3D-printed (m = 11.5), thus rejecting the null hypothesis that no differences exist between the two materials. The influence of the surface treatment was observed in both the milled and printed groups. Significant differences were observed after the milled PCs were sandblasted against the control (no treatment) and coarse diamond bur, while no differences were observed in the fine diamond bur surface treatment, thereby partially rejecting the null hypothesis for milled PCs. In the printed group, significant differences existed between sandblasting and all other groups (control, coarse, and fine diamond surface treatments), while coarse diamond surface treatment differed significantly from all three other groups. The fine diamond surface treatment did not differ significantly from the control, while it differed significantly from the coarse and sandblasting surface treatments. Barring a fine diamond, the null hypothesis for all other differences is therefore rejected. Both milled and 3D printing use the digital impression technology of scanning to record the details of either the surface anatomy of the tooth directed (intraoral scanners) or the surface of the impression and/or the dental cast. This provides multiple clinical and technical advantages related to storage, replication, transportation, and patient comfort [16,25]. The digital machining for both milled and 3D-printed parts has overcome the problem of processing errors that were incorporated in indirect restorations.

Influence of chemical constituents and polymer structures: The differences in the SBS between the milled and printed materials are primarily due to the differences in the compositions of the materials used. PMMA, Bis-GMA, and UDMA are light-curing resins used in 3D printing but have high shrinkage rates and poor mechanical properties. The dental photopolymerizable composition for 3D printers consists of a methacrylate-based polymerizable monomer with a urethane (NH—CO—O—) structure and a non-urethane structure, a filler (inorganic that is cohesive), and an initiator for polymerization (photo) [49] (Table 1). Urethane dimethacrylate (UEDMA) (7,7,9-trimethyl-4,13-dioxo-3, 14-dioxa-5, 2-diaza hexadecane-1,16-diyl dimethacrylate) forms the main constituent of PMMA 3D print inks and is a colourless liquid with a molecular weight of 470.6 g/mol [50]. Its chemical reactivity is because of its ability of donating two hydrogen bonds while accepting eight bonds at the same time. It also has multiple rotatable bonding ability that allows it to form a covalent bonded unit [49]. The base monomer concentration significantly impacts double-bond conversion, sol fraction, and crosslinking in printer inks [51]. Higher concentrations decrease double-bond conversion, increase the leachable fraction, and decrease crosslinking [52]. UDMA polymers have higher conversion, improved strength (flexure), and declined shrinkage [50]. Desirable properties of inkjet compositions include a balance between low viscosity, stability, thermal reactivity, and balanced thermoset and thermoplastic properties after curing [51]. The reactive species free radicals are produced when diphenyl phosphine oxide, a photoinitiator ranging from 0.1% to 5% by weight, is subjected to ultraviolet or visible light [50]. The ink’s viscosity can be affected or increased by cationic photoinitiators, which is why thermal cationic photoinitiators are better [49,51]. The mol. wt. of tetrahydrofurfuryl methacrylate (THFMA), another monomer (secondary), is 170.21 g/mol, making it a significant component of PMMA 3D printing ink [53]. Along with a rotatable and a covalently bound unit, it can absorb two hydrogen bonds but donate none. By default, it supplies a collection of molecules that could be useful in building the actual structure (canonical form). Ions are both very reactive but very transient [51]. Ionization occurs when a high-energy electron hits a molecule and knocks off one of its electrons, whether they are bonding or not. As a result, a molecular ion is left behind [51]. The molecular ion could break up into smaller, less energetic fragment ions and neutral bits due to the collision’s residual energy. The neutral fragment determines whether the fragment ions are radical cations or carbocations; the molecular ion is a radical cation [54]. Tetrahydrofurfuryl methacrylate is a suitable crosslinker for DLP 3D printing resin, improving rigidity, repairability, and re-processability [55]. Its incorporation leads to a 100% recovery ratio, improved tensile strength, and a higher recycling efficiency (344%). THFMA, derived from renewable resources, enhances tensile strength for re-processability efficiency calculation by chemically altering furfural from hemicellulose [56]. In dental restorative materials, inorganic filler is blended for strength improvement. However, using photopolymerizable inorganic particles in 3D printing results in excessive curing, lowering the modelling accuracy and limiting the attainment of excellent aesthetic properties. This is overcome by the addition of an ultraviolet (organic) absorber (≤500 nm) [49]. The dental photopolymerizable organisation for a 3D printer should have a branched structure with at least triple or more acrylate moieties, with 1 to 15 wt.% of the monomer consisting of these moieties [49]. The composition should also have a cohesive inorganic filler with 60 to 100 wt.% of SiO_2_ and 0 to 40 wt.% of ZrO_2_, with a specific surface area of 10 to 300 m^2^/g and a particle mean diameter of 1 to 15 μm [49]. The composition for 3D printers also includes an additive, a colouring material, ultraviolet absorber, polymerization inhibitor, and fluorescent agent. The additive should be 0.0001 to 2 pts. wt. based on the composition excluding the additive, ensuring optimal results [53]. The use of urethane-structured methacrylate enhances interactions with inorganic fillers, maintaining the surface appearance and enhancing the strength characteristics of modelled objects [49]. A polyfunctional acrylate-based polymerizable monomer with a branched structure accelerates surface curing, resulting in a smooth, attractive surface for modelled objects. Cohesive inorganic fillers (surface area 10 to 300 m^2^/g) offer high-quality provisional restorations with exceptional transparency and aesthetic properties [49,53]. A high specific surface area in a dental composition (photopolymerizable) for a 3D printer leads to high viscosity, making modelled objects difficult, while a low specific surface area facilitates sedimentation. The ideal particle diameter for a cohesive inorganic filler is 1–15 μm, with 1–10 μm being preferred. Particles smaller than 1 μm have small light scattering but high viscosity, potentially hindering modellability. Larger particles may hinder dental restorations with excellent aesthetic properties. A silane coupling agent, such as phenyl trimethoxysilane, dimethyl dimethoxysilane, and methyl trimethoxysilane, should ideally be surface-treated onto the inorganic cohesive filler [49].

On the other hand, a PMMA resin block for dental cutting (milling) may cause cracks, especially in large volumes. This reduces the mechanical strength of dental prostheses. The milled resin block consists of a polymer matrix and filler, which is mixed and polymerized before being cured [57]. However, cracks may occur in both cases, with differences in degree, which is overcome by blending organic–inorganic composite particles (metals or ceramics and their oxides) as a filler for dental resin blocks thus ensuring particle shape remains substantially spherical (1–50 μm) [57]. Spherical inorganic composite particles prevent air bubble formation at interfaces with resin matrix [58]. The PMMA CADCAM block uses inorganic or organic–inorganic composite (spherical filler) particles with an acrylic resin matrix. The average particle diameter is 1–50 μm, with 5–60% mass content, making it suitable for CAD/CAM resin blocks [57,59]. The resin matrix is a dispersion medium for organic–inorganic composite particles. It can be thermoplastic or thermosetting, but high-transparent resins are preferred for aesthetics. These are preferred due to their safety, high transparency, and easy refractive index control [57]. PMMA is preferred because of its ease in polymerizability. The curable composition polymerization method uses light energy (photopolymerization), chemical reaction between peroxide and accelerator, or thermal energy (thermal polymerization). Photopolymerization and thermal polymerization are preferred due to arbitrary timing and simple operation [57,60]. Each polymerization method has its own advantages/disadvantages that range from production to clinical use. The thermal polymerization ensures uniform polymerization from the inner to the outer surface, which minimizes polymerization shrinkage at the interfaces and prevents the development of porosity within the PMMA block. Porosity within printed resin adversely influences the mechanical properties (flexural strength, modulus, and impact strength); density (weight of the prosthesis); and overall clinical performance. High-pressure polymerization increases the mechanical strength and higher Mw polymers, while free radical polymerization increases the polymerization rate, propagation rate constant, and termination rate constant. Higher molecular weight polymers produced under pressure improve the flexural strength and modulus. Polymethyl methacrylate (PMMA) is also a highly effective optical polymer with a 92% visible light transmittance, superior to glass, and can withstand UV radiation and harsh outdoor conditions [37,40]. The optimal compounding quantity for a polymerization initiator is 0.01 to 5 mass parts in relation to 100 mass parts of resin matrices [57]. Silica-based composite oxide filler (40–800 nm) particles can easily adjust the refractive index and surface modification using silane coupling agents due to silanol groups [58]. To improve their wettability with the polymerizable monomer, the inorganic aggregated particles in organic–inorganic composites are often surface-treated with a hydrophobizing agent [60]. The cutting resin blocks also include optional components such as fillers (other than primary), polymerization initiators, inhibitors, fluorescent agents, ultraviolet absorbers, antioxidants, pigments, antibacterial agents, and X-ray contrast agents [57].

Differences in the SBS of orthodontic brackets have been observed for different restoration surfaces. Shirazi M et al. reported mean SBS values for an amalgam (6.55 MPa) to be lower than a composite (9.68 MPa) [61]. Likewise, there are differences in SBS values for different bracket materials (ceramic, metal, and polycarbonate) used against different restoration surfaces (ceramic, composite, natural tooth, and amalgam) [23,24,62]. The SBS values of orthodontic brackets on various restorations have been found to be lower when compared to natural teeth [6,16]. Our SBS values on 3D-printed (Asiga DentaTooth) PCs range from 11.5 MPa (no treatment) to 14.11 Mpa (sandblast), which are almost similar to those obtained by Choi Y et al. [63] in a recently concluded study using the same resin and bonding agent. However, in their study, they used ceramic brackets, and in one of their subgroups, they experimented with an unpolymerized liquid of printing resin as an adhesive, which yielded SBS values of 14.13 MPa. The results of their study also had the limitation of no thermocycling, but they reported using provisional crowns instead of disc specimens, which matches the convex contour of the bracket, which, in turn, increases the SBS. Our high SBS for printed resin are mainly attributed to the optimisation that was undertaken for printing, as described by Tahayeri A et al. [36]. Optimisation and calibration of the printer before printing according to the resin ink and the ink colour used increases the printing accuracy and improves both the mechanical and optical properties of the PC [16,36]. Sayed ME et al., in their study, reported the lack of 3D printer optimisation in studies that compared milled PMMA PCs with 3D-printed resin and stated that most of the studies had performed printing at the manufacturer settings (built-in programmed parameters) [16]. Optimising the printer for a particular resin ink was not mentioned in the study conducted by Choi Y et al. [63]. DentaTooth (Asiga, Alexandria, Australia) is used in the fabrication of various restorations (inlay, veneer, provisional crowns, and bridges) and prostheses (dentures and denture teeth). They undergo polymerization slowly in increments, which leads to free monomer and pigment accumulation in the structure, which gets released later, forming pores that decrease its physical properties. Methyl acrylate derivatives after curing have been reported to swell and form pores, allowing the further diffusion of monomers and pigments [64]. The results obtained for two different materials in this study are therefore in agreement with previous studies [1,6,17], which concluded that surface type has a significant influence on the bond strength of orthodontic stainless steel brackets. On the contrary, Goymen M et al. [2] did not find any influence of surface type on the bond strength between self-cured (Dentalon Plus, Basworth Trim II) and composite (Voco Structure Premium, Protemp, Revotek LC) resins. His study results were, however, at the end of 500 cycles, which clinically is equivalent to less than a month [three weeks] of time. PMMA 3D printing samples have been reported to exhibit significantly greater water sorption but without any change in solubility when compared to heat-cured PMMA resin [65]. Three-dimensional-printed resins with low polymerization degrees produce unreacted monomers, leading to high water sorption. Other constituents like crosslinking agents, plasticizers, initiators, and soluble materials also contribute to its higher water sorption properties. Post-thermocycling, the study also reports a stabilized water sorption within the PMMA print samples. Increased flexural strength in 3D print resin occurs during thermocycling, which is due to the presence of multiple monomers crosslinking between them and also due to the presence of mineral fillers, which prevents weakening through the development of cracks [66]. This explains the high SBS obtained in our specimens made of 3D printing material.

The high SBS values for milled resin in our study are also attributed to the basic composition (pre-polymerized PMMA blanks), which has been reported to have less porosity, more surface hardness, and less water sorption and solubility [16,45,67]. The pre-polymerized, prefabricated PMMA pucks (blocks) used in milling are manufactured with longer polymer chains, which helps them achieve higher free monomer conversion [18], resulting in less free monomer available. The amount of free monomer available post-polymerization is directly related to the hydrolytic degradation during thermocycling, thus explaining the maintenance of high SBS values after thermocycling [16,18]. Decreased hydrolytic degradation of milled PMMA resin is attributed to its low water sorption (0.03%), low water solubility (<0.8 μg/mm^3^), and low shrinkage rates (0.5%) [16,18,68]. These abilities are directly dependent upon the crosslinking chains between polymer and polymer. Other significant contributors to the improved performance of milled PMMA resin pucks are polymerization under high pressure and temperature and controlled monomer polymer parameters (ratio, temperature, and polymerization time) [68]. The results of the SBS averages in our study, however, contradict those obtained by Haber D et al. [39] and Goracci C et al. [40], who reported SBS between 2.71 and 5.35 and 5.23 and 5.95 MPa for milled PC material. The differences can be due to different manufacturers and the methods employed. Haber D et al. reported a reduction of SBS from the control [average 6.58 MPa] using Ceramill provisional CADCAM PMMA, while Goracci C et al. used two different PC materials [CAD-Temp (VITA) and Telio CAD (Ivoclar-Vivadent)]. Both studies also used different tools to simulate mechanical surface treatments on their PCs [diamond bur and sand paper].

Influence of Surface Treatment: Our study results show that mechanical surface treatments in the form of sandblasting and using a fine diamond bur increased SBS after thermocycling in both milled and printed PCs. The coarse diamond produced a decreased bond strength compared to the control in milled pieces, while remaining almost the same in printed PCs. Sandblasting is an effective way of improving retention of the adhesive and has been reported to increase the SBS for composites [1,6,17,22], polymethymethacrylate [1,2,7,17,21], polyethylmethacrylate [1], acrylic resin [1,21,69,70], and polycarbonate [7]. Higher SBS values have been reported than those obtained in this study for either milled or printed PCs. De Almeida JX et al. reported the SBS average with surface roughening [18.04 MPa] and sandblasting [22.64 MPa] in acrylic resins [duralay] [21]. Shahin SY reported higher SBS values with the sandblasting of composite PCs. The net differences between coarse and fine diamonds are in accordance with the findings of an earlier study, [22] which reported decreased SBS with coarse grit diamond burs when used on bisacrylic composites. The decreased bond strength attained by coarse diamond can be explained on the basis of the surface topography created and the residues left after using such an abrasive. Coarse diamond burs have been associated with the production of thicker smear layers on the dentin [71], which indicates that they leave a thicker layer of material residue in both 3D-printed and milled PCs. Since the layer is not removed before the application of the bonding agent, it would be worthwhile to investigate whether the removal of the residual layer before bonding to PCs would enhance the bond strength. Another possible reason for decreased bond strength associated with coarse diamond surface treatment is that coarse burs are more prone to dulling themselves during use. Both coarse and fine diamonds also produce a surface irregularity topography that is more linear, thereby leaving the surface between the irregular lines untreated. Hypothetically, this could be overcome by using both diamond burs in multiple directions so that the area that is left untreated between two lines can also be roughened. However, to prove this, one needs to conduct a study before reaching a conclusion. Contrary to its effects on various materials used for PCs, a study has reported that sandblasting (alumina oxide particles) did not affect the bond between the denture base (PMMA) surface and a resilient liner [2]. The sand particles at high speed penetrate and create the surface roughening by creating small valleys, while the surface that is not penetrated acts as a peak, thereby not only increasing the surface area but also creating a mechanical type of interlocking for the adhesive to stick to the substrate [17]. Since only one bonding agent (Assure Plus) and adhesive (Transbond XT) were used for all specimens in both material groups, the results on the bonding agent are slightly lower than those obtained on natural teeth, which have been reported to be 20.29 MPa and 18.45 MPa, respectively [72]. The bonding agent (Assure Plus) promotes adhesion mainly by their ingredients, which include monomers (10-methacryloyloxydecyl dihydrogenphosphate (10-MDP) and hydroxyethyl methacrylate (HEMA), which increase the flow by decreasing the viscosity while providing a chemical bond with the methacrylate group of the substrate [73]. Assure Plus bonding resin also combines BisGMA (10–30%) and ethanol (50–75%), promoting the infiltration of hydrophobic dimethacrylate resins into dentinal tubules and interfibrillar gaps in natural teeth.

Adhesive Remnant Index: One of the criteria for clinical acceptability of PCs is the amount of adhesive that is left over during removal. Low ARI scores allow clinicians to remove the bracket without leaving adhesive on the surface, saving clinical time that would otherwise be utilized to clean the surface. Low ARI scores also indicate that less damage will be incurred on the substrate (enamel and restoration) surface. The lowest mean ARI scores were observed in milled PCs as compared to printed PCs, with the lowest mean scores observed in GP MC (0.40) and GP MSB (0.80), while the highest were observed in the coarse diamond surface treatment in both milled [1.7] and printed [2.1] groups. When the ranks were analysed statistically, there was a significant difference in ranks between the studied subgroups. Differences in ARI scores were attributed to various influences that include the texture on the surface, degree of roughness, bracket surface (base) design, and surface chemistry between the material and the adhesive [74,75]. The basis of easy removal is the bond strength between the orthodontic bracket, the adhesive, and the surface of the PC. Lower ARI scores in the milled group indicate that the bond between the adhesive and the PC surface is slightly weaker than the bond between the orthodontic bracket and the adhesive. In no way does it indicate the nature of the strength of the entire bond.

Strengths and Limitations: The unique feature of this study is that it investigates the currently available two common digital technologies that have impacted dentistry in general. The study also stands apart due to its novelty and study design. While limited materials have been used, the study follows a very long duration of ageing that is clinically desirable in most cases. The study, despite having few strengths, does have its own limitations, which include a smaller number of brackets and adhesives investigated and fewer commercially available milled and printed PCs investigated. These limitations are mainly due to financial constraints; therefore, further studies are advised.

## 5. Conclusions

With similar prevailing in vitro conditions as in this study, the following can be concluded: that the SBS of orthodontic brackets varies with different PC materials—in this case, the materials being milled and printed PC materials; that both materials produced the clinically acceptable SBS for orthodontic brackets to PC (6 to 8 MPa) after different surface treatments (coarse, fine diamond, and sandblast); and that, after thermocycling (5000 cycles) that replicates the clinical time of almost 2 years, the SBS were still high for both digital crowns, although the printed group showed a decline when treated with a coarse diamond bur. The study also concludes that different surface treatments (both mechanical and chemical) produced different SBS, which are significantly different from each other and from those where no surface treatment was done. Based on the analysis of the ARI scores, the study results recommend that, for long-term orthodontic treatment, milled PCs can be used, since they permit easy debonding with less residual adhesive on the crown surface, which allows the clinician to remove the residue quickly.

## Figures and Tables

**Table 1:** List of materials, instrumentation, and manufacturers.

3D-print resin ink (PC)	Asiga DentaTooth Alexandria, NSW, Australia	Lot number: MO/16020 [Class II(a)] Photopolymerized Methacrylate resin: [7,7,9 trimethyl-4,13-dioxo3,14-dioxa-5,12diazahexadecane-1,16diyl bismethacrylate (urethane dimethacrylate)], tetrahydrofurfuryl methacrylate, and diphenyl phosphine oxide Post processing—light cure Colours: A1, A2, A3, B1, B2, B3 Curing: 4000 flashes (2 × 2000 flashes each side).
3D Printing Machine	Asiga Pty Ltd. Alexandria, NSW, Australia	Serial: 70B3D5362C6A Model: PN01233 Layer thickness 50 µm Post-Curing Machine—Asiga Flash (Wavelength: 405 nm)
CopraTemp	CopraTemp WhitePeaks Dental Solutions GmbH, Wesel, Germany.	Lot number: P10690 CADCAM PMMA blanks (shade A1); Plaque resistant Component: Polymethylmethacrylate, fillers (metals or ceramics and their oxides), additives, pigments Uses: long-term temporary restoration, splints, preparation guides and denture bases. Transparent/clear, classic pink, veined pink or precolored in the dentin colors A1, A2, A3 and B1
DGShape Five Axis milling machine	Roland DGA, Irvine, CA, Los Angeles, USA	DWX-52D Series (5-axis milling) Operating speed XYZ axis: 6 to 1800 mm/min. Spindle speed 6000 to 30,000 rpm Automatic disc changing mill
Thermocycling machine	Model 1100, SD Mechatronik, Bayern, Bavaria, Germany	Alternate immersion in warm followed by cold liquid simulates high temperature changes Warm bath temperature: 25 °C to 55 °C Cold bath temperature: 5 °C to 15 °C Exposure time—30 s, open air—10 s
Benchtop 3D Scanner	MEDIT Model MD-ID0300, Medit Corp, Seoul, Republic Korea	Scans full arch in 8 s. 5.0 MP cameras
Imageing powder	VITA CEREC, VITA, Bad Sackingen, Germany	Powder scan spray
Assure Plus	Reliance Orthodontic Products, Thorndale Ave, Itasca, IL 60143, Itasca, IL, USA	All Surface Bonding Resin (light cure) Bis-GMA, ethanol, MDP, HEMA
Transbond XT	3M Unitek, Monrovia, CA 91016, USA	Light cure adhesive paste Silane treated quartz (70–80% in weight), bisphenol A diglycidyl ether dimethacrylate, bisphenol A bis(2-hydroxyethyl ether) dimethacrylate, silane treated silica, diphenyliodonium

**Table 2:** Comparative differences in the means of shear bond strengths between orthodontic brackets to milled (CopraTemp) and 3D-printed (Asiga) provisional restorative materials after surface treatment and ageing.

	Variable 1		Variable 2—Surface Treatment				Analysis of Variance Tests			
	Material	Parameters	No Treatment (*n* = 10)	Coarse Diamond (*n* = 10)	Fine Diamond (*n* = 10)	Sandblast (*n* = 10)	Independent One Way ANOVA		Interaction Two Way ANOVA	
							F Statistic	*p* Value	F Statistic (df_1_, df_2_)	*p* Value
Shear Bond Strength (MPa)	MILLED (Gp M) (N = 40)	Sub Group	MC	MCD	MFD	MSB	33.86	0.00001 *	3.7961 (3, 72)	0.01381 *
		M	13.24	11.28	13.76	15.51				
		SD	0.94	0.53	1.26	0.89				
	3D-printed (Gp P) (N = 40)	Sub Group	PC	PCD	PFD	PSB	11.2518	0.00002 *		
		M	11.5	11.62	12.76	14.11				
		SD	1.8	1.25	1.22	0.87				

Abbreviations: MPa = megapascals; N = number of specimens; M = mean (in MPa); SD = standard deviation. Group description/coding: M = milled, P = printed, MC = milled control, MCD = milled coarse diamond, MFD = milled fine diamond, MSB = milled sandblast, PC = printed control, PCD = printed coarse diamond, PFD = printed fine diamond, and PSB = printed sandblast. Test employed: one-way analysis of variance (ANOVA) for differences within groups and two-way ANOVA for differences between groups. Statistical significance: All differences at various time intervals in each group were considered to be statistically significant if the probable *p*-value was ≤0.05. Symbol * = statistically significant.

**Table 3:** Tukey’s HSD (Honestly Significant Difference) post hoc pairwise comparison showing the overall interactive differences in the sample means between milled (CopraTemp) and 3D-printed (Asiga) provisional restorative materials after various mechanical surface treatments.

Subgroups	MC	MCD	MFD	MSB	PC	PCD	PFD	PSB
MC		1.962	0.526	2.277	1.741	1.62	0.525	0.87
		0.002 *	0.9504	0.0001 *	0.009 *	0.020 *	0.950	0.587
MCD	1.962		2.488	4.239	0.221	0.342	1.437	2.832
	0.002 *		0.0000 *	0.0000 *	0.999	0.995	0.058	0.0000 *
MFD	0.526	2.488		1.751	2.267	2.146	1.051	0.344
	0.9504	0.0000 *		0.008 *	0.0001 *	0.0005 *	0.342	0.995
MSB	2.277	4.239	1.751		4.018	3.897	2.802	1.407
	0.0001 *	0.0000 *	0.008 *		0.0000 *	0.0000 *	0.0000 *	0.069
PC	1.741	0.221	2.267	4.018		0.121	1.216	2.611
	0.009 *	0.999	0.0001 *	0.0000 *		1.000	0.177	0.0000 *
PCD	1.62	0.342	2.146	3.897	0.121		1.095	2.49
	0.020 *	0.995	0.0005 *	0.0000 *	1.000		0.291	0.0000 *
PFD	0.525	1.437	1.051	2.802	1.216	1.095		1.395
	0.950	0.058	0.342	0.0000 *	0.177	0.291		0.0738
PSB	0.87	2.832	0.344	1.407	2.611	2.49	1.395	
	0.587	0.0000 *	0.995	0.069	0.0000 *	0.0000 *	0.0738	

Note: Abbreviations: HSD = Honestly Significant Difference; Group description/coding: MC = milled control, MCD = milled coarse diamond, MFD = milled fine diamond, MSB = milled sandblast, PC = printed control, PCD = printed coarse diamond, PFD = printed fine diamond, and PSB = printed sandblast. Statistical significance: All differences between various subgroups were considered to be statistically significant if the probable *p*-value was ≤0.05. Symbol * = statistically significant.

**Table 4:** One-way ANOVA on ranks (Kruskal–Wallis), non-parametric test showing the differences between the mean rank scores of various subgroups of milled (CopraTemp) and 3D-printed (Asiga) provisional restorative materials for the adhesive remnant index (ARI) scores.

Groups	Subgroups	N	df	Median	Mean Rank Scores	H Test Statistic	*p* Value
MILLED (Gp M) (N = 40)	MC	10	7	0	17.4	26.54	0.00040 *
	MCD	10	7	2	51.75		
	MFD	10	7	1	38.9		
	MSB	10	7	0.5	29.1		
3D-printed (Gp P) (N = 40)	PC	10	7	1	41.85		
	PCD	10	7	2	59.35		
	PFD	10	7	2	50.2		
	PSB	10	7	1	35.45		

Note: Abbreviations: Gp = group; N = number; df = degree of freedom; *p* = probability. Group description/coding: M = milled, P = printed, MC = milled control, MCD = milled coarse diamond, MFD = milled fine diamond, MSB = milled sandblast, PC = printed control, PCD = printed coarse diamond, PFD = printed fine diamond, and PSB = printed sandblast. Statistical significance: All differences between various subgroups were considered to be statistically significant if the probable *p*-value was ≤0.05. Test employed: Kruskal–Wallis test, using chi-squared (df: 7) distribution (right-tailed). Symbol * = statistically significant.

**Table 5:** Post hoc Dunn’s test using Bonferroni correction showing the overall interactive differences in the sample medians between various subgroups of milled (CopraTemp) and 3D-printed (Asiga) provisional restorative materials for the adhesive remnant index (ARI) scores.

Subgroups	MC	MCD	MFD	MSB	PC	PCD	PFD	PSB
MC		−34.35	−21.5	−11.7	−24.45	−41.95	−32.8	−18.05
		0.0004 *	0.0279	0.2316	0.0124	0.00001 *	0.00079 *	0.0649
MCD	−34.35		12.85	22.65	9.9	−7.6	1.55	16.3
	0.0004 *		0.1889	0.02058	0.3115	0.4372	0.8741	0.0956
MFD	−21.5	12.85		9.8	−2.95	−20.45	−11.3	3.45
	0.0279	0.1889		0.3164	0.763	0.0365	0.248	0.7243
MSB	−11.7	22.65	9.8		−12.75	−30.25	−21.1	−6.35
	0.2316	0.02058	0.3164		0.1924	0.00198	0.03099	0.5162
PC	−24.45	9.9	−2.95	−12.75		−17.5	−8.35	6.4
	0.0124	0.3115	0.763	0.1924		0.07359	0.3933	0.5129
PCD	−41.95	−7.6	−20.45	−30.25	−17.5		9.15	23.9
	0.00001 *	0.4372	0.0365	0.00198	0.07359		0.3495	0.01455
PFD	−32.8	1.55	−11.3	−21.1	−8.35	9.15		14.75
	0.00079 *	0.8741	0.248	0.03099	0.3933	0.3495		0.1316
PSB	−18.05	16.3	3.45	−6.35	6.4	23.9	14.75	
	0.0649	0.0956	0.7243	0.5162	0.5129	0.01455	0.1316	

Abbreviations: Group description/coding: MC = milled control, MCD = milled coarse diamond, MFD = milled fine diamond, MSB = milled sandblast, PC = printed control, PCD = printed coarse diamond, PFD = printed fine diamond, and PSB = printed sandblast. The corrected α using the Bonferroni correction method is 0.001786 [Corrected α = α/m = 0.05/28 = 0.001786]. Symbol * = statistically significant.

## Data Availability

All relevant data have been presented within the article; however, the raw data files are available from the corresponding author and can be available upon reasonable request.
