# Correction: Hassan et al. Influence of Surface Treatment and Protracted Ageing on the Shear Bond Strength of Orthodontic Brackets to Two Digitally Fabricated (Milled and 3D-Printed) Polymethacrylate-Based Provisional Crowns. *Polymers* 2025, *17*, 699

**DOI:** 10.3390/polym18040472

**Published:** 2026-02-13

**Authors:** Nisreen Nabiel Hassan, Khurshid Mattoo, Atheer Khawaji, Hanan Najmi, Almaha Sadeli, Ahid Amer Alshahrani, Abeer Ali Qahtani, Abdullah Hasan Alshehri, Mai Almarzouki, Mohammed E. Sayed

**Affiliations:** 1 Department of Restorative Dental Sciences, College of Dentistry, Taibah University, Madinah 41477, Saudi Arabia; nnhassan@taibahu.edu.sa; 2 Department of Prosthetic Dental Sciences, College of Dentistry, Jazan University, Jazan 45142, Saudi Arabia; kmattoo@jazanu.edu.sa; 3 Intern Clinic, College of Dentistry, Jazan University, Jazan 45142, Saudi Arabia; atheer.khawajix2@gmail.com (A.K.); hanan.najmi111@gmail.com (H.N.); sadeli.almaha@gmail.com (A.S.); 4 Department of Dental Technology, College of Applied Medical Sciences, King Khalid University, Abha 62529, Saudi Arabia; aalshahrani1@kku.edu.sa; 5 Department of Restorative Dental Science, Taif University, Taif 26571, Saudi Arabia; abeer.a.qahtani1@gmail.com; 6 Department of Prosthodontics, College of Dentistry, King Khalid University, Abha 62529, Saudi Arabia; abhalshehri@kku.edu.sa; 7 Department of Restorative Dentistry, Faculty of Dentistry, King Abdulaziz University, Jeddah 21589, Saudi Arabia; mzalmarzouki@kau.edu.sa

**Error in Figure**

In the original publication [1], there was a mistake in Figure 1 as published. The corrected Figure 1 appears below. The authors state that the scientific conclusions are unaffected. This correction was approved by the Academic Editor. The original publication has also been updated. This figure was included by mistake and should have been replaced with the actual picture.

## Figures and Tables

**Figure 1:**
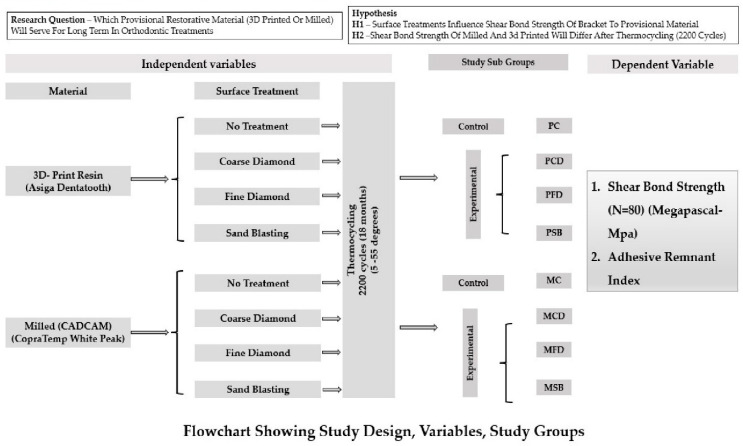
Flowchart showing the overall study design, the independent and dependent variables, and the study groups.

## References

[1] Hassan N.N., Mattoo K., Khawaji A., Najmi H., Sadeli A., Alshahrani A.A., Qahtani A.A., Alshehri A.H., Almarzouki M., Sayed M.E. (2025). Influence of Surface Treatment and Protracted Ageing on the Shear Bond Strength of Orthodontic Brackets to Two Digitally Fabricated (Milled and 3D-Printed) Polymethacrylate-Based Provisional Crowns. Polymers.

